# MicroRNA as a Prognostic and Diagnostic Marker in T-Cell Acute Lymphoblastic Leukemia

**DOI:** 10.3390/ijms22105317

**Published:** 2021-05-18

**Authors:** Katarzyna Gębarowska, Anna Mroczek, Jerzy R. Kowalczyk, Monika Lejman

**Affiliations:** 1 Laboratory of Genetic Diagnostics, Medical University of Lublin, 20-093 Lublin, Poland; katgebarowska@gmail.com; 2 Department of Pediatric Hematology, Oncology and Transplantology, Medical University of Lublin, 20-093 Lublin, Poland; anna.mroczek94@wp.pl (A.M.); jerzy.kowalczyk@uszd.lublin.pl (J.R.K.)

**Keywords:** T-ALL, microRNA, T-ALL markers, diagnostic marker, predictive marker

## Abstract

T cell acute lymphoblastic leukemia (T-ALL) is a biologically and genetically heterogeneous disease with a poor prognosis overall and several subtypes. The neoplastic transformation takes place through the accumulation of numerous genetic and epigenetic abnormalities. There are only a few prognostic factors in comparison to B cell precursor acute lymphoblastic leukemia, which is characterized by a lower variability and more homogeneous course. The microarray and next-generation sequencing (NGS) technologies exploring the coding and non-coding part of the genome allow us to reveal the complexity of the genomic and transcriptomic background of T-ALL. miRNAs are a class of non-coding RNAs that are involved in the regulation of cellular functions: cell proliferations, apoptosis, migrations, and many other processes. No miRNA has become a significant prognostic and diagnostic factor in T-ALL to date; therefore, this topic of investigation is extremely important, and T-ALL is the subject of intensive research among scientists. The altered expression of many genes in T-ALL might also be caused by wide miRNA dysregulation. The following review focuses on summarizing and characterizing the microRNAs of pediatric patients with T-ALL diagnosis and their potential future use as predictive factors.

## 1. Introduction

**Figure 1:**
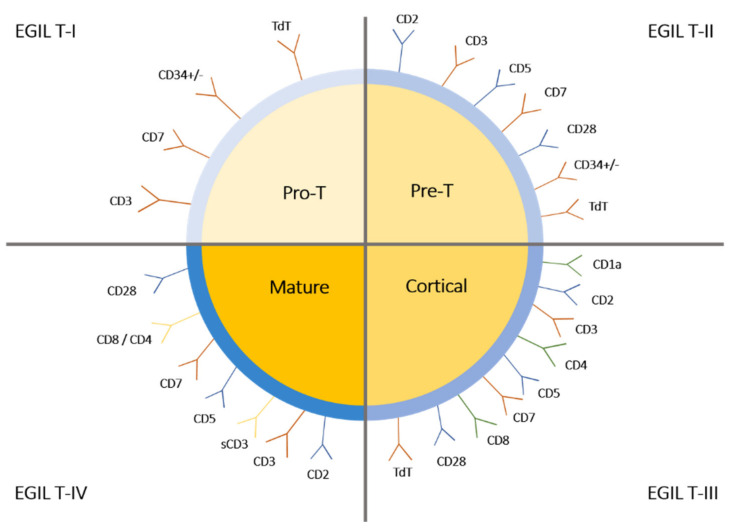
The European Group for the Immunologic Classification (EGIL) of T-ALL.

Pediatric acute lymphoblastic leukemia (ALL) is an aggressive type of cancer originating from the lymphoid lineage that accounts for 80% of all pediatric leukemia [1]. ALL occurs with an approximate frequency of 1.5 out of 100,000 people, of which ~25% of adult and ~12% of pediatric patients suffer from T cell acute lymphoblastic leukemia (T-ALL) [1,2,3]. T-ALL is a biologically and genetically heterogeneous disease with a poor prognosis overall and several subtypes that are distinguished by the expression of surface and intracellular antigens. According to The European Group for the Immunologic Classification (EGIL) of T-ALL, four subgroups are distinguished based on the immunophenotype of T cells: the EGIL T-I subset refers to pro-T and is characterized by the presence of CD3, CD7, TdT, and CD34 (+/−); in the EGIL T-II subgroup—pre-T—additional CD2, CD5, and CD28 antigens are expressed; the third subset contains cortical T cells, which are additionally CD1a positive and CD4/CD8 double positive; finally, EGIL T-IV is represented by mature T cells, where CD3 and CD4 or CD8 are present on the cell surface, while CD1a and TdT are no longer observed (Figure 1) [4,5].

Early T cell Precursor ALL (ETP-ALL) is classified as one of the T-ALL subtypes with a high rate of treatment failure. ETP-ALL presents a unique immunophenotype and a specific genetic profile, similar to hematopoietic stem cells and myeloid progenitors, with a high expression of self-renewal genes, including LMO2/LYL1, HOXA, and BCL2 [6,7]. ETP-ALL occurs in 15% of pediatric and 35% of adult patients (Table 1) [8,9,10].

The molecular basis of T-ALL includes chromosomal rearrangements and mutation responsible for the activation of oncogenes or inactivation of tumor-suppressor genes. These abnormalities involve: transcription factors: TAL1, TLX1, TLX3, HOXA, NKX2-1, LMO1-2/LYL1; Notch pathway: NOTCH-MYC-FBXW7; cell-cycle regulation: CDKN2A and CDK4/6 complex; kinase signaling: IL7R, JAK1/3, STAT5, and PI3K/AKT/mTOR; RNA metabolism/translation: RPL5, RPL10, RPL11, RPL22, CNOT3 complex, and EIF4A [4,10,11,12,13,14].

The altered expression of many genes in T-ALL might also be caused by wide miRNA deregulation. A large number of microRNAs (miRNAs) have been reported to have an important role in the development and pathogenesis of almost all human cancers, including hematological malignancy [15,16]. The microarray and next-generation sequencing (NGS) technologies exploring the coding and non-coding part of the genome allow us to reveal the complexity of the genomic and transcriptomic background of T-ALL. DNA methylation, histone modifications, and aberrant miRNA expression have been shown to play a role in leukemogenesis in children and the progression of leukemia in adults [15,16]. miRNAs are a class of non-coding RNAs that are involved in the regulation of cellular functions: cell proliferations, apoptosis, migrations, and many other processes. The following review focuses on summarizing and characterizing the microRNAs of pediatric patients diagnosed with T-ALL and their potential future use as predictive factors.

## 2. microRNA in T-ALL

microRNAs (miRNAs) are short (21–23 nucleotides), endogenous, noncoding single-strand RNAs molecules with gene expression regulation capability [17]. miRNAs often function together as co-regulating groups for the same cellular process, as exemplified by clusters of miRNAs. They are either transcribed as a primary polycistronic transcript or structurally unrelated but co-expressed and functionally related to each other. Such clustered and co-expressed miRNAs cause a phenotypic effect, e.g., oncogenic or tumor suppressor effects. miRNAs are involved in transcription, translation, and epigenetic regulation, and their structure prediction and target molecules can be provided by various bioinformatic tools [18]. miRNAs function generally as endogenous translational repressors of protein-coding genes through sequence-specific binding to the 3′ untranslated region (3′UTR) of a target messenger RNA. Moreover, it is estimated that they may affect the expression of up to 60% of genes encoding proteins [19]. The aberrant expression of miRNAs can function as either oncogenes or tumor suppressors in T-ALL. miRNAs are presumed to be involved in oncogenesis and are currently the subject of researchers’ interest as potential prognostic factors and therapeutic targets [20]. miRNAs could also be potential markers, which would improve the classification of T-ALL patients and provide more effective targeted therapy. Differentiation between ALL subtypes may be possible by using miRNAs, as some of the above-mentioned subtypes present different miRNA expression profiles [21]. According to research conducted by Coskun et al., the downregulation of miR-708 and upregulation of miR-196b were present more often in patients with T-ALL than in other groups [20]. The discussed studies also indicate that a higher expression of miR-196b correlates with a higher incidence of an immature stage of T cell immunophenotypes [22,23]. A few studies describe different miRNA expression profiles between ETP-ALL and non ETP-ALL cases. Coskun et al. showed that miR-221 exhibited an eight times higher expression (*p* < 0.01) and five times higher miR-222 expression in ETP-ALL compared to non-ETP-ALL cases. Research shows that miR-222 and miR-221 overexpression (OE) can induce chemo-resistance and worse 5-year overall survival (OS) rates, respectively [24,25,26]. Additionally, it was indicated that high expression of miR-363 and miR-19a was associated with a more mature T-ALL phenotype [24]. The differences between normal lymphocytes and T-ALL were also evaluated in a study conducted in 2017 by a team of researchers led by Wallaert. Moreover, miR-128a and miR-181b were overexpressed while miR-100 and let-7e downregulation (DR) was observed in the T-ALL group in comparison to the control group [27].

### 2.1. The Influence of miRNA on the Development of T-ALL

The miRNA expression profile may be useful for the assessment of the estimated survival time and response to treatment in T-ALL patients [25]. Nemes et al. investigated the relationship between treatment responses with ALL and miRNA expression. The results of the research revealed a significantly poor prognosis and poor response to prednisone treatment on day 8 in ALL patients with reduced miR-128b expression. Researchers reported an increase in the disease-free period in the miR-128b overexpression group compared to the group with miR-128b downregulation. Patients were characterized during their relapse by a low level of miR-223 and increased expression of miR-128b [28]. Hypermethylation of mir-124 was related to the unfavorable development of the disease through increased mortality and recurrence rates and the shorter OS and disease-free survival (DFS). The results were found to be statistically significant [29]. miR-100 and miR-99a have been reported to be aberrantly expressed in ALL, and lower expression levels of both miR-100 and miR-99a were found in T-ALL patients and patients carrying the *MLL*-rearrangement or *BCR-ABL1* fusion genes, and these lower expression levels correlated with a poor prognosis. These results indicate that the expressions of miR-100 and miR-99a are cell-type specific and suggest that both miRNAs are associated with leukemogenesis and the prognostic outcome of ALL [30]. Luo and others have shown that the overexpression of miR-429 can be responsible for T-ALL progression. Additionally, miR-1246/1248 OE was associated with the recurrence of the disease [31].

### 2.2. The Impact of miRNA on the Response to Treatment

Resistance to treatment is most often acquired. One of the reasons for this is that miRNAs regulate the expression of proteins responsible for therapy failure. This allows the cancer cells to gain the desired feature [32]. Of the total number of miRNAs, five were found to be most important in cross-resistance to drugs (miR-9-5p, miR-125b-5p, miR-130b-3p, miR-199b-5p, and miR-200c-3p) [32]. The downregulation of miR-210 was associated with cross-resistance to daunorubicin, dexamethasone, and asparaginase, causing reduced OS and DFS and an additionally increased risk of relapse [33]. Low miR-101 expression was found to enhance the effect of NOTCH1 and induce cancer cell insensitivity to doxorubicin [34]. The upregulation of miR-100-5p, miR-125b-5p, and miR-331-3p was observed in patients with unsatisfactory results to vincristine treatment [32]. Both Drobna et al. and Schotte et al. have noticed that interactions between the enhanced expressiveness of miR-100, miR-99a, and miR-125b led to vincristine and daunorubicin resistance [23,26].

### 2.3. The Most Common miRNAs in T-ALL and Their Prognostic Potential

**Figure 2:**
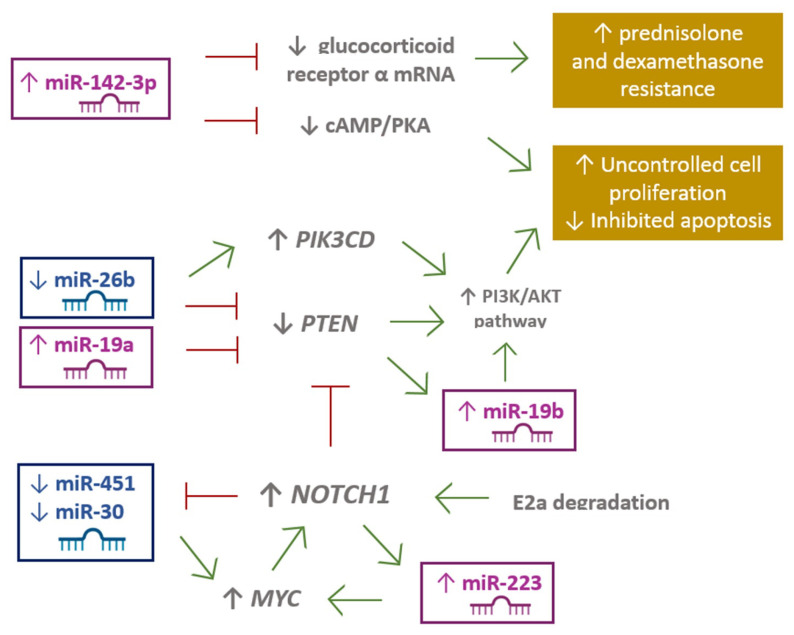
miRNA expression effects on signaling pathways in T-ALL.

The 10 most frequently observed miRNAs in T-ALL patients are miR-16, -19b, -20a, -26a, -92, -93, -142-3p, -150, -223, and miR-342 [25,35]. These miRNAs act as oncogenes by influencing the suppressor genes involved in T-ALL leukemogenesis, such as *BIM*, *FBXW7*, *IKZF1*, *NF1*, *PTEN*, and *PHF6* [26]. Furthermore, they also contribute to the pathogenesis of T-ALL through *NOTCH* signaling dysregulation [25,26]. A role in the pathogenesis of T-ALL has also been assigned to miR-128-3p, -148a-3p, -181a/b, -363-3p, and 20b-5p [20,21]. Altered expression of different miRNAs affects a variety of signaling pathways, including the NOTCH1 pathway, resulting in uncontrolled cell proliferation, inhibition of apoptosis, as well as drug resistance (Figure 2).

#### 2.3.1. miR-17-92 Cluster

The miRNAs cluster mir-17-92 can be transcribed into seven mature miRNAs-miR-17-5p, -17-3p, -18a, -19a, -20a, -19b, and -92, and their increased expression is observed in patients with T-ALL [25,36]. The coding region is located at chromosome 13q31. This cluster is involved in chromosome abnormalities, including the translocation t(13;14)(q32;q11) with the *TCRA/D* locus in T-ALL [35]. miR-19b shows oncogenic activity in patients with T-ALL induced by *NOTCH1* through the inhibition of the *PTEN*, *CDKN1A*, and *BCL2L11* suppressor genes [20,35]. The *PI3K* pathway strongly correlates with increased cell survival, and its higher activation was observed in miR19a overexpression. Similarly, to miR-19b, the overexpression of miR19a leads to the inhibition of the suppressor genes *BIM (BCL2L11)*, *PRKAAA1* (AMP-activated kinase), *PP2A* (*PPP2R5E*), and *PTEN* (tumor suppressor phosphatases). The same conclusions were reached by Coskun and Correia [24,25]. Additionally, Coskun and others observed a correlation between a low expression of miR-19a and increased *IGFBP7*, *MN1*, and *WT1* in bone marrow (BM) aspirates from T-ALL patients. Researchers in this group demonstrated that the inhibitory effect on *NOTCH1*-induced T-ALL progression was obtained using the antagomir miR19 [24]. Mavrakis et al. also confirmed that the coexpression of 19a/b, 26a (targeting *PTEN*, *PHF6*), and miR-92 in the T-ALL-1 cell line was strongly related to T-ALL progression compared to a single overexpression [35].

#### 2.3.2. miR-106a-363 Cluster

The miR-106a-363 cluster encoding six miRNAs (miR-106a, miR-18b, miR-20b, miR-19b, miR-92a, and miR-363) is a paralogue of the oncogenic miR-17-92a polycistron, and its role in leukemia is at present largely unknown. The analyses performed in T-ALL cells in vitro indicate that the miR-106a-363 cluster may have an oncogenic role in the pathogenesis of T-ALL via the suppression of pro-apoptotic genes [20,26].

#### 2.3.3. miR-221-222 Cluster

The importance of miR-221 and miR-222 in the development of many types of cancers has been described in numerous research works, proving an association between a higher expression of miR-221/miR-222 and poorer overall survival rates [37]. Furthermore, the miR-221-222 cluster has been found to play an important role in T-ALL development and therapy response. Coscun et al. compared miRNA expression profiles in bone marrow (BM) aspirates from ETP-ALL and non-ETP-ALL adult patients. Significant upregulation, specific for ETP-ALL, was observed for miR-221 and miR-222. They also noticed a higher expression of miR-222 in ETP-ALL than in acute myeloid leukemia (AML); meanwhile, no significant difference was observed for miR-221. Both miR-221 and miR-222 were remarkably more highly expressed in ETP-ALL in comparison to healthy mature CD3+ cells. Furthermore, they identified the target gene of miR-222: the protooncogene ETS1. ETS1 is accountable for apoptosis regulation, and its inactivation can lead to the defective activation of T-cells. They also suggested that the altered expression of *ETS1* induced by miR-222 might contribute to the myeloid character of ETP-ALL [24]. Gimenes et al. showed a higher expression of miR-221 in the peripheral blood cell CD56+ than in CD56- in T-ALL; additionally, a higher level of miR-221 was observed in leukemic blasts in comparison to normal T-cells and thymocytes. It was also suggested that the high expression of both miR-221 and miR-222 is required to maintain a continuous capability to proliferate by targeting cell cycle regulators, such as CDKN1B/p27^Kip1^ and c-KIT receptor. P27^Kip1^ modulates the platelet-derived growth factor (PDGF) signaling pathway, which was reported to be upregulated in cytotoxic T and NK cell neoplasms [38]. Shao-Wu et al. found miR-221 in significantly higher amounts in the peripheral blood mononuclear cells (PBMNCs) of children with T-ALL than in a control group. In the study, patients’ samples were differentiated into three groups: initial group, samples taken before treatment and then samples after 33 days, depending on achieving complete remission. Samples were divided into refractory or remission groups. As a result, significantly higher expression levels of miR-221 were observed in the refractory group than in the initial or remission group. Therefore, a high level of miR-221 was found to correlate with poorer response to induction therapy, contributing to the development of minimal residual disease. The research also showed a correlation with the expression level of miR-221 and blood cell count and risk typing, making miR-221 a promising diagnostic as well as prognostic factor [39].

#### 2.3.4. miR-16

mir-16 is recognized as a cancer suppressor with proapoptotic properties and can be considered as a potential prognostic biomarker in T-ALL. The dysregulation of miR-16 has already been observed in a study conducted by Gao et al. on K562 and HL60 cell lines, which are models of chronic myelogenous leukemia (CLL) and Caucasian promyelocytic leukemia, respectively. It was observed that increased expression of miR16-1 efficiently inhibited cell proliferation [40]. Similar results were obtained by Aqeilan et al., where somatic cell hybrids between mouse LM-TK^−^ and CLL cells carrying 13q14.3 translocations or deletions were generated [41]. Xiaoqin et al., considering isolated mouse peritoneal macrophages, suggested that miR-16 can mediate the PD-1/PD-L1 signaling pathway, which is involved in the activation and regulation of T cell apoptosis, by directly or indirectly downregulating PD-L1 expression [42]. A study of lymph node samples from T-ALL patients showed that there was no significant difference in expression levels between T-ALL patients (n = 72) and the control group with reactive lymph nodes (n = 17); nonetheless, it has been observed that a higher expression level of miR-16 was a good prognostic factor. The overall one-year survival rate for the entire cohort was 38.9%; however, the miR-16 high-expression group was characterized by significantly higher overall survival, as the one-year survival was almost twice as high as that in the low-expression group, with rates of 50% and 2.5% respectively [43].

#### 2.3.5. miR-21

The role of miR-21 has been extensively investigated in carcinogenesis. A study conducted by Junker et al. focused on the diagnostic and prognostic value of miR-21 as well as its implication in the drug resistance of human malignancies. A study in murine models of NOTCH-driven T cell leukemia showed that miR-21 influences tumor suppressor genes associated with proliferation, apoptosis, and invasion. A microarray test indicated that miR-21 was deregulated in human T cells. The results also showed that miR-21 influences the repression of the tumor suppressor PDCD4 via the induction of apoptosis [44].

#### 2.3.6. miR-26b

In the same manner, as miR-26b, the dysregulation of miR-26b shows a similar effect in T-ALL development. miR-26b acts as a tumor suppressor, and its expression is closely associated with *PTEN* activation. PTEN functions as a repressor of the PI3K/AKT pathway, the constitutive hyperactivation of which was observed in more than 87% of T-ALL patients’ peripheral blood and bone marrow samples [45]. Both enhanced apoptosis and the inhibition of proliferation of T-ALL cells, as well as an impeded progression of T-ALL disease, can be achieved by the expression of exogenous miR-26b. Yuan et al. showed that miR-26b inhibits the PI3K/AKT signaling pathway by the repression of the *PIK3CD* gene. shRNA knockdown of *PIK3CD* and CAL-101—two PIK3CD inhibitors—also promoted the apoptosis of T-ALL cells. Additionally, loss of *PTEN* activity in the mice cell line leads to a significantly decreased expression of miR-26b [46].

#### 2.3.7. miR-30

The role of miRNA-30 as a suppressor has been suggested. Its low expression was observed in patients with a T-ALL diagnosis. A mutual correlation between the *MYC*-protooncogene used to activate cell division and miR-30 transcription and NOTCH1 and 2 genes were observed. It is worth mentioning that *MYC* inhibits the expression of miR-30, which is directed towards and inhibits the *NOTCH1* and *NOTCH2* genes. Moreover, a positive correlation was found with *NOTCH1*, which activates the transcription of *MYC* [26]. Furthermore, a study conducted by Drobna et al. showed that low expression of miR-30a-5p in mononuclear cells from bone marrow samples of T_ALL pediatric patients leads to an increase in STK39 (serine/threonine kinase 39) levels involved in the development of leukemia [21].

#### 2.3.8. miR-100

Xue et al. conducted a case-control study to evaluate the relationship between genetic variants in miR-100, miR-146a, and miR-210 and the risk of childhood ALL in the Chinese population. Based on blood samples from children and adolescents, they suggested that there was a significant association between the polymorphisms in miR-100 (rs543412) and decreased susceptibility to childhood ALL [47]. Hassan et al. searched for a difference in miR-100 expression between the following ALL types: pre-B-ALL, B-ALL, and T-ALL. miR-100 expression was measured in samples from the bone marrow aspirate of 85 pediatric ALL patients and 12 healthy donors. Their results revealed miR-100 overexpression in B-ALL; meanwhile, in T-ALL, miR-100 was significantly downregulated. They suggested that low expression of miR-100 could be associated with high-risk prognostic factors and was also found in patients carrying BCR-ABL1 fusion genes or MLL rearrangement. miR-100 was also significantly associated with shorter overall survival and disease-free survival rates, making miR-100 a potential prognostic marker [48].

#### 2.3.9. miR-124a

In over 50% of both pediatric and adult T-ALL, underexpression of miR-124a is observed. A decrease in miR-124a level is a result of promoter hypermethylation. The cascade of reactions starts from the downregulation of miR-124a, which leads to cyclin-dependent kinase 6 (*CKD6*) overexpression. CKD6 is responsible for retinoblastoma protein (RB1) phosphorylation; therefore, there is a reduction of the RB1 active form, resulting in enhanced proliferation and apoptosis inhibition [26].

#### 2.3.10. miR-125b and miR-99a

mir-125b, along with miR-99a, is highly upregulated in the specific T-ALL subtype homeobox 3 positive T-ALL (TLX3). Renou et al. showed that a high expression of TLX3 and miR-125b in thymic cells enhances the production of T cell progenitors, increases the invasiveness of T-ALL, and contributes to the accumulation of immature T cells. It has been shown that, due to the transactivation of the long noncoding RNA gene *LINC00478*, TLX3 is capable of regulating both miR-99a and miR-125b, resulting in increased T cell proliferation. The oncogenic activity of miR-125b can be inhibited through TLX3 knockdown by designed short hairpin RNA (shRNA) transfection [49].

#### 2.3.11. miR-128a

miR-128a can be characterized as both a suppressor and an oncogene. Oncogenic action is based on the demethylation of its promoter region and suppression of *FADD* and *PHF6* genes, which have an effect on the inhibition of FAS-mediated apoptosis and tumor suppression, respectively. Additionally, the overexpression of miR-128a is observed in NOTCH1-induced T-ALL. On the other hand, miR-128a has a suppressive effect in mixed lineage leukemia ALL (MLL, new name *KMT2A*) development [26]. In a study conducted by Carvalho et al., miR-128a was observed more frequently in samples from the bone marrow of childhood ALL patients. Additionally, a higher expression of miR-128a is supposed to be associated with t(4;11) ALL [50].

#### 2.3.12. miR-142-3p

miR-142 plays a major role in T-ALL progression. The overexpression of this small RNA molecule leads to downregulation of the *cAMP/PKA* pathway, which leads to increasing T-ALL cell proliferation with no influencing apoptosis. Moreover, miR-142-3p targets mRNA coding glucocorticoid receptor alpha (GRα). Through reduced receptor GRα biosynthesis, this leads to T-ALL cell resistance to prednisolone [25,26]. Studies in the T-ALL cell line have shown that the inhibition of miR-142-3p may cause increased sensitivity to steroid therapy [26]. Additionally, in a study based on the analysis of peripheral blood from T-ALL patients, Lv et al. observed that in patients with prednisolone resistance, the inhibition of miR-142-3p causes improved sensitivity to dexamethasone [25,51]. The above results suggest that miR-142-3p antagomir treatment may apply to potential future therapies in T-ALL patients.

#### 2.3.13. miR-146b-5p

Tumor suppressive activity in T-ALL patients is also represented by miRNA-146b-5p. The overexpression of miRNA-146b-5p results in the inhibition of migration, invasiveness, and metastasis of cancer cells and is the result of positive regulation by *TAL1* [26]. Reduced MiR-146b-5p expression was found in T-ALL cell lines in comparison to normal T cells [52,53]. The results suggest that low expression of this miRNA by increasing levels of Il-17 and metalloproteinase 9 (MMP9) stimulates T-ALL cells for migration and infiltration [52]. Correia et al., through a comparison of miR-146b-5p levels between T-ALL primary cells, T-ALL cell lines, normal T-cells, thymocytes, CD34+ hematopoietic progenitor cells, and bone marrow precursors, confirmed that TAL1+ patients had lower miRNA-146b-5p levels. A study on mouse models showed that the expression of miR-146b-5p leads to better results in the form of increased OS. Low expression of miRNA-146b-5p was associated with shorter OS, worse prognosis, and a higher risk of metastasis to the central nervous system [53].

#### 2.3.14. miR-150

Another miRNA that plays a key role in hematopoietic development is miR-150. Its lower expression was observed in patients with T-ALL; therefore, it is concluded that this miR-150 has a suppressive effect on cancer development. In the research based on the LeukemiR database, Rawoof et al. presumed targets of miR-150 that were two tumor suppressors, *SMAD4* and *NOTCH3*, the downregulation of which leads to enhanced TGF-β and NOTCH signaling pathways, respectively. As a result, hematopoietic progenitor growth and proliferation are significantly increased [36]. It has also been observed that miR-150 interacts with another crucial protein, MYC, the overexpression of which negatively regulates miR-150 levels [26].

#### 2.3.15. miR-193b-3p

Higher levels of miR-193b-3p were observed in healthy donors’ thymocyte cells as compared to T-ALL. A lower expression of miR-193b-3p was shown to be characteristic for the *TAL*-rearranged T-ALL subtype. Similar results were obtained for NOTCH1-induced T-ALL. miRNA-193b-3p is a putative tumor suppressor as it is involved in the inhibition of two protooncogenes: *MYB*, which is responsible for cell proliferation, and *MCL1*, which affects resistance to apoptosis. The regulation of *MYB* and *MCL1* is based on site-specific complementarity [54].

#### 2.3.16. miR-196b

In pediatric patients with T-ALL, miR-196b interaction with the *HOXA* gene cluster has been reported. This interaction could be due to its location in the human genome: miR-196b is mapped between the *HOXA9* and *HOXA10*, and therefore its coregulation is HOXA dependent. Increased proliferation and apoptosis inhibition of progenitor cells is a result of the overexpression of miR-196b in bone marrow [50]. In mouse models, it has a role in the process of oncogenesis by giving neoplastic cells the desired characteristics, such as higher survival and proliferative capacity [25]. Another study showed the inhibition of cell growth in *KMT2A-MLLT3* (formerly, *MLL-AF9*) transformed bone marrow cells after the application of miR-196a antagonist. High expression of miR-196b specifically occurred in *KMT2A*-rearranged and T-ALL patients carrying *CALM*-*AF10* or *SET*-*NUP214* fusions and an inversion of chromosome 7. These molecular and morphological changes are functionally linked with the upregulation of HOXA [22,55]. A reversed relationship was observed in adult patients in which miRNA inhibited the transcription of oncogenic ERG protein, thus exhibiting features of a tumor suppressor [25]. Additionally, lower expression of miR-196b in comparison to normal cells was observed in pediatric patients, which may indicate the tumor suppressor properties of this miRNA [25,26]. Compared to B cell precursor acute lymphoblastic leukemia (BCP-ALL), the T-ALL pediatric group was characterized by higher miR-196b expression [50].

#### 2.3.17. miR-223

miR-223 is another important miRNA for the pathogenesis of T-ALL. miR-223 is believed to have an oncogenic effect on T cell development by reducing tumor suppressor genes, such as *E2F1*, *FOXO1*, *RHOB*, or *EPB41L3.* Moreover, it has been proven that miR-223, by regulating the E3 ligase FBXW7, has an antiapoptotic activity through MCL1 growth [25]. There is evidence that *TAL1,* through the interaction between miR-223 and *FBXW7,* regulates the level of *MYC* transcription [25]. In a study conducted on several types of T-ALL cell lines as well as samples from patients by Mansour et al., it was observed that increased levels of miR-223 through high TAL-1 expression led to a decrease in FBXW7 expression. This interaction also leads to an increase of MYC, MYB NOTCH1, and cyclin E [56]. T-ALL patients were characterized by a high expression of miR-223-5p and low expression of miR-223-3p, the targets of which were *GAB1*, *STAT3*, and *IL6ST,* respectively [21]. Similar results were obtained in the work by Nemes et al. They suggested that the low expression of miR-223 detected in peripheral blood and bone marrow samples from ALL children was associated with the risk of T-ALL recurrence [28]. It is worth noting that in a study carried out by Gusscott’s research team, the human T-ALL cell lines that were treated with γ-secretase inhibitors (GSI) were characterized by a higher miR-223 level, which suggests the negative regulation of miR-223 expression through NOTCH1 [57].

#### 2.3.18. miR-326

Ghodousi et al. searched for miRNA that might affect the expression of brain and acute lymphoblastic leukemia (*BAALC*). The study was based on the analysis of bone marrow samples from children with ALL with controls and CCRF-CEM cell lines. Their analysis showed overexpression of *BAALC* with a simultaneous downregulation of miR-326 in drug-resistant ALL. The *BAALC* gene is expressed in the nervous system, as well as in the bone marrow hematopoietic precursor cluster of CD34+ cells. Its overexpression is associated with poor outcomes in children with ALL, therapy resistance, leukemogenesis, shorter survival, and MRD. In the study, they suggest that the high expression of *BAALC* combined with low expression of miR-326 may be an independent prognostic marker in children with ALL due to the characteristic expression profile in MRD and relapsed pediatric patients [58].

#### 2.3.19. miR-451

It has been shown that miR-451 plays a crucial role in many types of cancers, and its important function has been described in a variable sort of solid tumors. Its altered expression in the form of downregulation can also be observed in leukemias [59,60]. Jiang et al., in their study based on human T-ALL cell xenografts in mice, showed that miR-451 can act as a gene suppressor and that it is closely related to the NOTCH1 signaling pathway. NOTCH1’s downstream target is *MYC*, the dysregulation of which is essential in T-ALL [61]. T-ALL cells induce *NOTCH1* through the reaction cascade (with E2a degradation), leading to a decrease in miR-451 expression. The downregulation of miR-451 causes an increase in *MYC* expression, which promotes the proliferation of neoplastic cells [62,63].

#### 2.3.20. miR-664

Another overexpressed miRNA observed in pediatric T-ALL is miR-664. miR-664, through the intermediary of *PLP2* gene inhibition, alters the ability for cell adhesion and migration through an increased amount of surface CD99 protein [26]. It has also been experimentally proven on an osteosarcoma cell line that the inhibition of miR-664 decreases cell growth, inhibits cells’ migration capability, and promotes apoptosis [64].

#### 2.3.21. mir-708

mir-708 might be used as another prognostic marker to estimate the relapse-free survival (RFS) time of patients, as its dysregulated expression was observed in childhood ALL relapse. A study of paired bone marrow samples with 8 diagnosis-relapse and 10 diagnosis-complete remission samples revealed that, in diagnosis samples, miR-708 was the most upregulated miRNA among all tested miRNAs. In samples from patients after complete remission, the mir-708 level was significantly decreased in comparison to diagnosis and relapse samples. Furthermore, the therapy response was substantially different in high and low miR-708 level samples; it was discovered that a good response for prednisone was obtained in patients with high levels of miR-708 at initial diagnosis; meanwhile, in the group with a poor response to prednisone, detected miR-708 levels were lower. Moreover, patients considered as having a standard and medium risk showed significantly higher miR-708 levels than high-risk patients, which therefore implies that miR-708 at a low expression level corresponds with a higher risk of relapse. Furthermore, among the four types of ALL—pre B-ALL, pro-B-ALL, common ALL, and T-ALL, T-ALL was the one with the lowest miR-708 expression levels [65]. On the other hand, a study carried out on several types of ALL cell lines—mouse model, 21 patients with T-ALL, and 58 patients with B-ALL—by Huang et al. showed that miR-708 negatively regulates CD47 expression and plays a suppressive role in T-ALL development. CD47 is responsible for the inhibition of phagocytosis and is observed to be highly expressed in T-ALL in contrast to B-ALL. The upregulation of CD47 in T cells enables cells to avoid phagocytosis and is observed in parallel with a decrease in miR-708. It is believed that miR-708 could be a potential medicine and could be used in T-ALL CD47-targeted therapy [66]. We have listed different types of miRNAs that are up-/downregulated in T-ALL cases and associated with a response to the treatment or outcome (Table 2).

## 3. Conclusions

miRNAs are associated with the regulation of normal hematopoiesis, and miRNAs can function as oncogenes and tumor suppressors whose deregulation is associated with the development and progress of many types of cancers. Research is increasingly focused on the molecular basis of diseases, including altered miRNA expression. There is an increasing body of evidence that miRNA profiling can contribute to improved patient classification and the use of targeted therapy suitable for known drug response. In general, works in the literature confirmed the significant impact of miRNA expression levels in leukemogenesis in pediatric and adult T-ALL. Moreover, this can imply the potential of miRNAs to be considered as therapeutic targets or therapeutic tools for the prevention or regression of leukemia. Molecular profiling, based on miRNA, is not yet widespread in clinical practice, due to a lack of validation of thresholds and the ambiguity of data. Thus, more research and evidence-based meta-analyses of miRNA expression levels in different subtypes of T-ALL are required to develop better clinical opportunities for patients.

## Figures and Tables

**Table 1:** Immunophenotypic characterization of early T cell precursor acute lymphoblastic leukemia (ETP-ALL).

Genes overexpressed in ETP-ALL	*CD44, CD34, KIT, GATA2, CEPBA, SPI1, ID2, MYB*
Genes underexpressed in ETP-ALL	*CD1, CD3, CD4, CD8, RAG1, NOTCH3, PTCRA, LEF1, TCF12, LAT, LCK, TCF7, ZAP70*
Myeloid or stem cell markers	CD117, CD34, HLA-DR, CD13, CD33, CD11b, CD65
Immature phenotype of T cells in ETP-ALL	Lack of CD1a and CD8, weak CD5(dim) expression

**Table 2:** Summary of dysregulated miRNAs that are associated with a response to the treatment or outcome in patients with T-ALL.

miRNA	Locus	OE/DR	Subtype	Target	Outcome	Reference
miR-16	13q14.2	OE	T-ALL	No data	Higher one year OS	[43]
miR-17-5p	13q31	DR	T-ALL	Upregulation of lysosomal proteins	Poor prognosis Daunorubicin resistance	[32]
miR-26a	3p22.2	OE	T-ALL	*PHF6, PTEN*	Oncogenic role in T-ALL	[35]
miR-30a-3p	6q13	OE	T-ALL	*STAG2, PSME3, RAD1, PSMD11, PSMD7, PSMD3, MDM2, CDC27, CDK1, CCNA2*	Suppression of apoptosis by impair cell cycle arrest in G1 phase (*STAG2, PSME3, RAD1*)	[32]
miR-100	11q24.1	OE	T-ALL	No data	Vincristine resistance	[32]
miR-124a	8p23.1	DR	T-ALL	*CDK6;* phosphorylation of retinoblastoma	Vincristine resistance; Increased mortality and recurrence rates and the shorter OS, DFS; poor prognosis	[29]
miR-125b	11q24.0	OE	T-ALL	No data	Vincristine and daunorubicin cross-resistance	[32]
miR-125b-5p	11q24.1	OE	T-ALL	*MAD4*	Bortezomib and dexamethasone resistance	[32]
				*ETS1*	Vincistrine resistance	
miR-128b	3p22.3	DR	T-ALL	No data	Poor prognosis and poor response to prednisone treatment on day 8	[28]
miR-128-3p	2q21.3	DR	T-ALL	Upregulation of lysosomal proteins	Poor prognosis Daunorubicin resistance	[32]
miR-142-3p	17q22	OE	T-ALL	Decreased cAMP/PKA, GRa	Promote leukemic cell growth and glucocorticoid resistance	[51]
miR-146a-5p	5q33.3	OE	T-ALL	*STAG2, PSME3, RAD1, PSMD11, PSMD7, PSMD3, MDM2, CDC27, CDK1, CCNA2*	NGS signaling (*PSMD7*)	[32]
miR-221	Xp11.3	OE	ETP-ALL	No data	Lower 5-year OS; poor prognosis	[26]
				High (N-CAM; CD56)		[25]
miR-222	Xp11.3	OE	ETP-ALL	Protooncogene ETS1-inhibition	Poor prognosis	[24,25]
miR-223-3p; miR-223-5p	Xq12	DR	T-ALL	*IGFR*	Cell survival and proliferation (relapse)	[28]
				Increases IL6/JAK/STAT	No data	[21]
				*PTEN, BIM, NF1, FBXW7, IKZF1, PHF6*	Increases the level of anti-apoptotic MCL1	
				Decreases: *E2F1*, *FOXO1*, *RHOB*, *EPB41L3*	No data	
		OE		FBXW7; MYC	No data	[25,35]
miR-326	11q13.4	OE	T-ALL	*BAALC*	Drug resistance	[48]
miR-363-3p	Xq26.2	DR	ETP-ALL	High MN1	No data	[24]
miR-451a	17q11.2	DR	*NOTCH1* T-ALL	Degradation E2a	T-ALL induced by *NOTCH1*	[62]
				Overexpression of *MYC*	Disease progression, increased T-ALL cell proliferation	
miR-708	11q14.1	OE	T-ALL	*E2F1*	No data	[65]

## Data Availability

No new data were created or analyzed in this study. Data sharing is not applicable to this article.

## Abbreviations

3′UTR	3′untranslated region
ALL	acute lymphoblastic leukemia
AML	Acute myeloid leukemia
BAALC	brain and acute lymphoblastic leukemia
BCP-ALL	B-cell precursor acute lymphoblastic leukemia
CKD6	cyclin dependent kinase 6
DFR	disease free of recurrence
DFS	disease-free survival
DR	downregulation
EGIL	The European Group for the Immunologic Classification
ETP-ALL	Early T-cell precursor ALL
GRα	glucocorticoid receptor alpha
GSI	γ-secretase inhibitors
JAK	Janus kinase
LBL	lymphoblastic lymphoma
miRNA	MicroRNA
MMP9	metalloproteinase 9
MRD	minimal residual disease
OE	overexpression
OS	overall survival
PBMNC	peripheral blood mononuclear cells
PDGF	Platelet Derived Growth Factor
RB1	retinoblastoma protein
RFS	relapse-free survival
shRNA	short hairpin RNA
STK39	serine/threonine kinase 39
T-ALL	T-cell acute lymphoblastic leukemia
TLX	T-cell leukemia homeobox 1
TLX3	homeobox 3
